# Identification of Candidate Genes and Regulatory Competitive Endogenous RNA (ceRNA) Networks Underlying Intramuscular Fat Content in Yorkshire Pigs with Extreme Fat Deposition Phenotypes

**DOI:** 10.3390/ijms232012596

**Published:** 2022-10-20

**Authors:** Yueyun Ding, Yinhui Hou, Zijing Ling, Qiong Chen, Tao Xu, Lifei Liu, Na Yu, Wenliang Ni, Xiaoling Ding, Xiaodong Zhang, Xianrui Zheng, Wenbin Bao, Zongjun Yin

**Affiliations:** 1 College of Animal Science and Technology, Anhui Agricultural University, Hefei 230036, China; 2 Anhui Province Key Laboratory of Local Livestock and Poultry Genetic Resource Conservation and Bio-Breeding, Anhui Agricultural University, Hefei 230036, China; 3 Key Laboratory for Animal Genetics, Breeding, Reproduction and Molecular Design of Jiangsu Province, College of Animal Science and Technology, Yangzhou University, Yangzhou 225009, China

**Keywords:** non-coding RNAs, ceRNA network, IMF, pig, RNA-seq

## Abstract

Intramuscular fat (IMF) content is vital for pork quality, serving an important role in economic performance in pig industry. Non-coding RNAs, with mRNAs, are involved in IMF deposition; however, their functions and regulatory mechanisms in porcine IMF remain elusive. This study assessed the whole transcriptome expression profiles of the *Longissimus dorsi* muscle of pigs with high (H) and low (L) IMF content to identify genes implicated in porcine IMF adipogenesis and their regulatory functions. Hundreds of differentially expressed RNAs were found to be involved in fatty acid metabolic processes, lipid metabolism, and fat cell differentiation. Furthermore, combing co-differential expression analyses, we constructed competing endogenous RNAs (ceRNA) regulatory networks, showing crosstalk among 30 lncRNAs and 61 mRNAs through 20 miRNAs, five circRNAs and 11 mRNAs through four miRNAs, and potential IMF deposition-related ceRNA subnetworks. Functional lncRNAs and circRNAs (such as MSTRG.12440.1, ENSSSCT00000066779, novel_circ_011355, novel_circ_011355) were found to act as ceRNAs of important lipid metabolism-related mRNAs (*LEP*, *IP6K1*, *FFAR4*, *CEBPA*, etc.) by sponging functional miRNAs (such as ssc-miR-196a, ssc-miR-200b, ssc-miR10391, miR486-y). These findings provide potential regulators and molecular regulatory networks that can be utilized for research on IMF traits in pigs, which would aid in marker-assisted selection to improve pork quality.

## 1. Introduction

Pork is the most widely consumed meat worldwide, accounting for 40% of human red meat consumption, and its quality directly affects human health [1]. Intramuscular fat (IMF) content is an important characteristic of pork quality and is positively associated with its palatability, marbling score, tenderness, juiciness, and overall meat quality [2]. It is generally accepted that a higher IMF content tends to be an indicator of high-grade pork [3]. Therefore, breeding pigs with more IMF can produce more palatable pork. With relevance to meat quality in pig and human health, IMF content is now an important economic trait in pig breeding programs [4,5].

In pigs, IMF has high heritability, with estimated values varying from 0.21 to 0.86 and an approximate average of 0.5 [6,7]. This high heritability makes porcine IMF a suitable and important indicator for selection breeding programs focused on obtaining high-quality pork. However, the direct selection of IMF content by traditional breeding methods during pig breeding is extremely difficult to implement, partly because of phenotyping complexity and partly because it is influenced by different factors, such as species, breed, age, sex, and nutritional levels [8]. Molecular breeding methods could be a valid method for swine IMF improvement; therefore, identifying potential molecular markers for IMF is now an important task in genetic research and pig molecular breeding programs [4,5,6,7]. Moreover, pigs are an ideal model for human obesity-related research because they share many similarities with humans at the physiological and genomic levels [9]. Therefore, studies on the molecular mechanisms of IMF deposition are important for improving the economic efficiency of pigs and human health.

As a polygenic trait, IMF is a complex metabolic process determined by the hyperplasia and hypertrophy of adipocytes and involves many biological processes and pathways, which are regulated by various molecules, such as hormones, transcription factors, protein-coding genes, and non-coding RNAs [10,11,12]. However, when compared with other traits, dissecting the genetic basis of IMF content is still limited, owing to its complexity. Previous findings have shown that porcine intramuscular adipogenesis is regulated not only by protein-coding RNAs (mRNAs) but also by non-coding RNAs (ncRNAs). For example, miR-146a-5p targets *SMAD4* and *TRAF6* to inhibit porcine intramuscular preadipocytes adipogenesis through the TGF-β and AKT/mTORC1 signaling pathways [13]. Further, LMFlnc1 (lncRNA) promotes adipogenesis in porcine IMF cells by upregulating *CAV-1* expression via miR-199a-5p [14]. Moreover, Sus_circPPARA promotes differentiation and hinders proliferation in porcine intramuscular preadipocytes [11]. Despite the increasing number of transcriptomic studies aimed at dissecting IMF deposition, cascade events related to this trait remain mostly unknown. Furthermore, non-coding RNAs related to IMF in pigs are largely unknown, and the mechanisms by which they regulate IMF deposition are unclear and require further study. Therefore, to fully elucidate the complex gene networks and molecular cascade pathways that regulate IMF deposition in pigs, it is crucial to expand basic knowledge by further characterizing coding and non-coding transcriptomes.

Transcriptome studies comparing individuals with extreme phenotypes of a trait are useful in identifying gene pathways and networks with divergent expression among groups of livestock species. IMF content can vary from 2 to 10% in different swine breeds [15,16]. For example, it is typically higher in indigenous Chinese pig breeds than in Western pig breeds and commercial pigs [17]. Pig breeds in China have high contents of subcutaneous and intramuscular fat and good meat quality [18]. However, commercial pig breeds, such as Duroc, Landrace, and Yorkshire, are selected for better lean muscle growth, leading to a decrease in the deposition of fat, including IMF, resulting in reduced meat palatability [12]. Studies have also shown that IMF varies considerably among pure commercial breeds [7,11,16]. For example, Yorkshire pigs are a typical lean-type Western breed and have a low level of fat deposition as a whole. However, phenotypic variation in IMF content is still observed in this population [16]. Previous transcriptome studies using pigs of different breeds have provided relevant results related to the genetic expression patterns and networks underlying IMF production [17,19]; however, few studies have been conducted on individuals of the same breed with distinct IMF contents to identify consistent candidate genes [15,16]. Improving IMF production from a genetic perspective in lean pigs is one of the major goals of pig breeding programs to improve meat quality, and it would be particularly interesting to analyze changes in the transcriptome and regulatory factors of finishing lean pigs with divergent IMF content.

In this study, we collected tissues of the *longissimus dorsi* muscle of castrated finishing Yorkshire males of similar ages and body weights, but with divergent back fat thickness and IMF. Whole-transcriptome sequencing of this tissue was performed to explore the gene profiles and identify candidate lncRNAs, circRNAs, miRNAs, and mRNAs associated with IMF deposition by evaluating differences in expression and identifying the pathways in which the differentially expressed genes are involved. Furthermore, potential lncRNA/circRNA–miRNA–mRNA co-regulatory networks were constructed to elucidate the complex genetic architecture related to swine IMF. This research could provide a comprehensive and bioinformatic resource to study the regulatory mechanisms of pig IMF deposition mediated by non-coding RNAs. These results might also contribute to improvements in pork quality at the genetic and molecular levels and provide insight into human obesity and related diseases.

## 2. Results

### 2.1. Characterization of the Longissimus Dorsi Transcription

**Figure 1:**
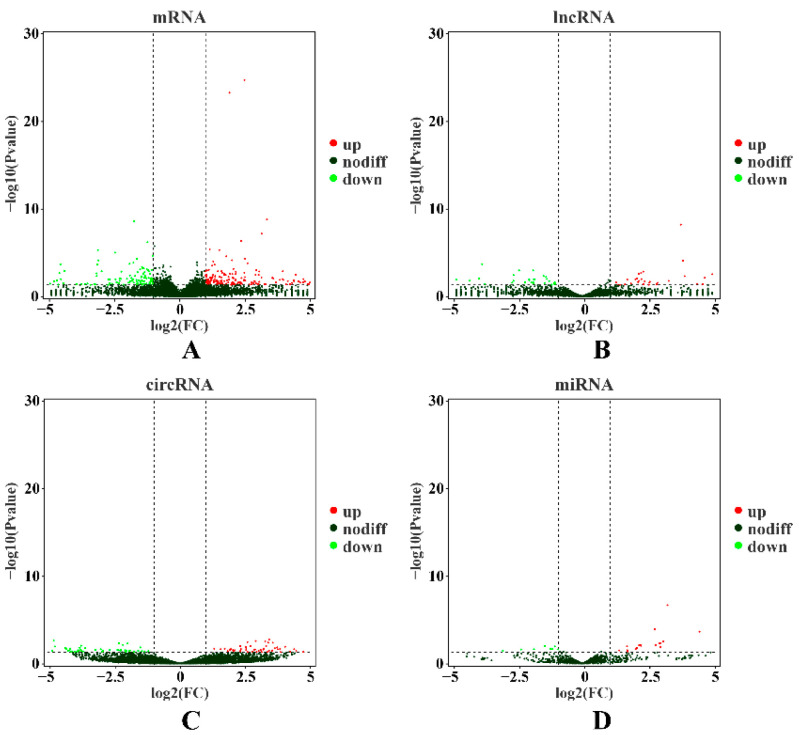
The DE mRNAs (**A**), lncRNAs (**B**), circRNA (**C**) and miRNAs (**D**) in the H vs. L groups. The vertical dotted lines indicate |log2FC| = 1, and the horizontal dotted lines indicate *p* value = 0.05.

Two cDNA libraries, an rRNA-depleted library and an miRNA library, were constructed. After the redundant and low-quality reads were removed, the rRNA-depleted library contained 82,402,852, 74,367,466, 124,581,402, 90,505,406, 12,780,608, and 137,892,896 clean reads with greater than 93.05% Q30 scores from the H1–3 and L1–3 samples, respectively, which were used to identify the mRNAs, lncRNAs, and circRNAs (Table S2). Among them, 94.94%, 95.94%, 95.69%, 95.86%, 93.86%, and 95.76% of reads from H1–3 and L1–3, respectively, were mapped to the pig reference genome (S. scrofa 11.1; Table S2). In addition, for the small RNASeq libraries, there were 32,998,980 raw reads (H: 9,685,832–12,734,725 for each library) and 34,161,122 raw reads (L: 9,295,100–13,349,696 for each library) in the H and L groups, respectively. After trimming and filtering, 32,168,194 (H) and 33,271,882 (L) clean reads were obtained. In total, 2,127,279 (H) and 2,143,330 (L) known miRNA reads and 23,953 (H) and 23,846 (L) novel miRNA reads were obtained after a series of analyses (Table S3). In total 21,353 mRNAs, 1088 miRNAs, 9980 lncRNAs, and 13,521 circRNAs were obtained from the H and L groups. Further, 341 mRNAs, 36 miRNAs, 91 lncRNAs, and 178 circRNAs were identified as differentially expressed (DE) RNA molecules between the two comparison groups (Table 1 and Figure 1A–D).

### 2.2. Transcriptional Profiling of mRNAs

**Figure 2:**
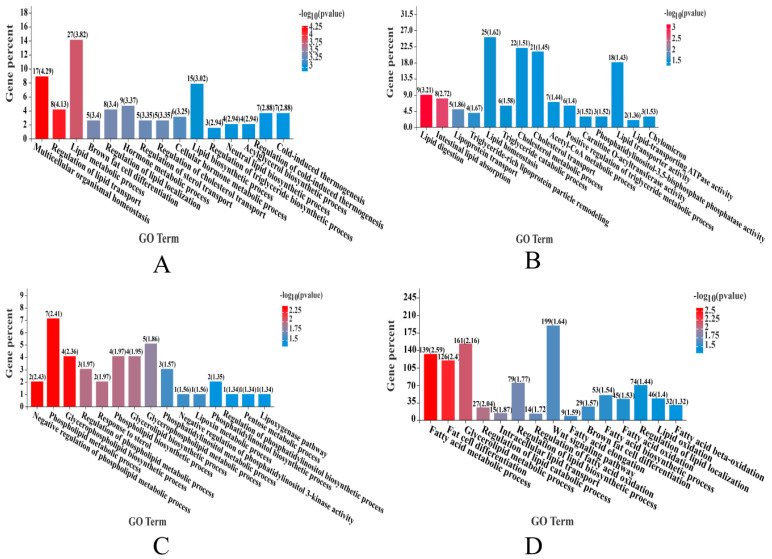
**A** Gene Ontology (GO) enrichment analysis. (**A**) GO analysis of DEMs in the H vs. L groups. (**B**) GO analysis of DE LncRNAs (DELs) in the H vs. L groups. (**C**) GO analysis of DE CircRNAs (DECs) in the H vs. L groups. (**D**) GO analysis of DE miRNAs (DEMiRs) in the H vs. L groups.

**Figure 3:**
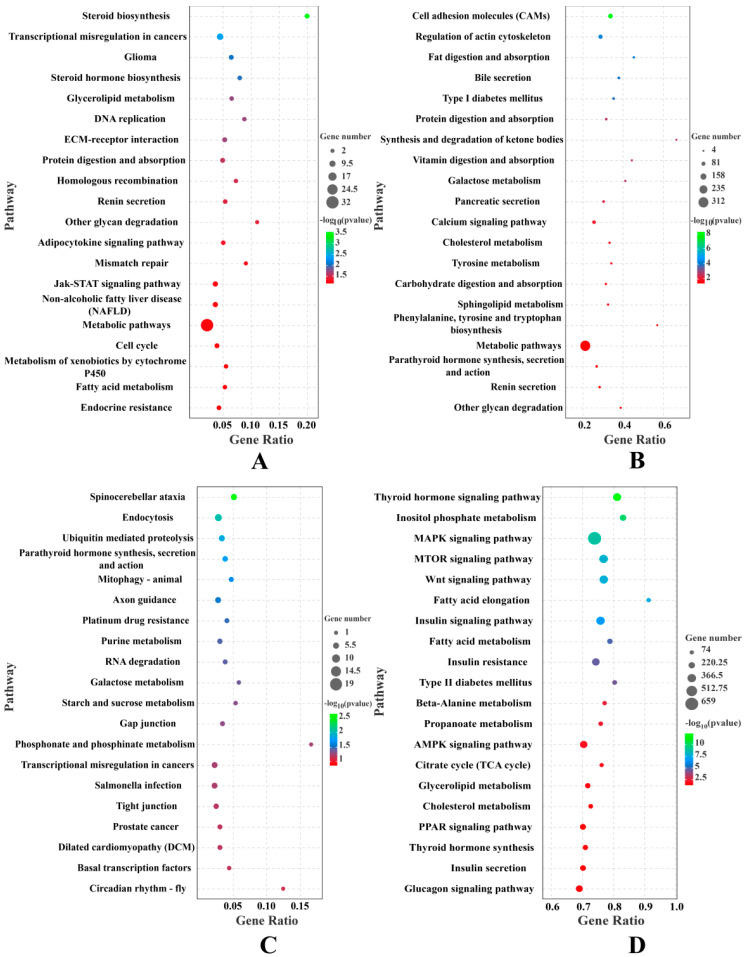
A KEGG analysis of DE genes in the H vs. L groups. (**A**) KEGG analysis of DEMs. (**B**) KEGG analysis of DELs. (**C**) KEGG analysis of DECs. (**D**) KEGG analysis of DEMiRs.

In the H vs. L group set analyses, 341 DE mRNAs (DEMs) were detected, with expression levels of 134 mRNAs downregulated and those of 207 mRNAs upregulated in the H group (Table 1 and Supplementary File S1), including a series of DE genes directly related to the regulation of IMF deposition, such as *ADIPOQ* (log2FC = −1.325), *CEBPA* (log2FC = −1.173), *ALOX12B* (log2FC = −3.624), *LEP* (log2FC = −3.148), *ACACA* (log2FC = −1.226), *DGAT2* (log2FC = −1.608), *ACLY* (log2FC = −6.554), *IP6K1* (log2FC = −7.907), *PLIN1* (log2FC = −1.759), *CYB5R1* (log2FC = −1.508), *ACBD7* (log2FC = 2.336), *SPP1* (log2FC = 2.864), *ADAMTS8* (log2FC = 2.921). GO analysis revealed that these DEMs were significantly enriched (*p* < 0.05) in terms related to fatty acid biosynthesis and lipid metabolism, such as the lipid metabolic process (GO:0006629), brown fat cell differentiation (GO:0050873), white fat cell differentiation (GO:0050872), regulation of cholesterol transport (GO:0032374), fatty acid metabolic process (GO:0006631) (Figure 2A, Table 2, and Supplementary File S1). KEGG analysis revealed that these DEMs were significantly enriched (*p* < 0.05) in steroid biosynthesis, steroid hormone biosynthesis, glycerolipid metabolism, adipocytokine signaling pathway, and steroid hormone biosynthesis (Figure 3A and Supplementary File S1).

**Figure 4:**
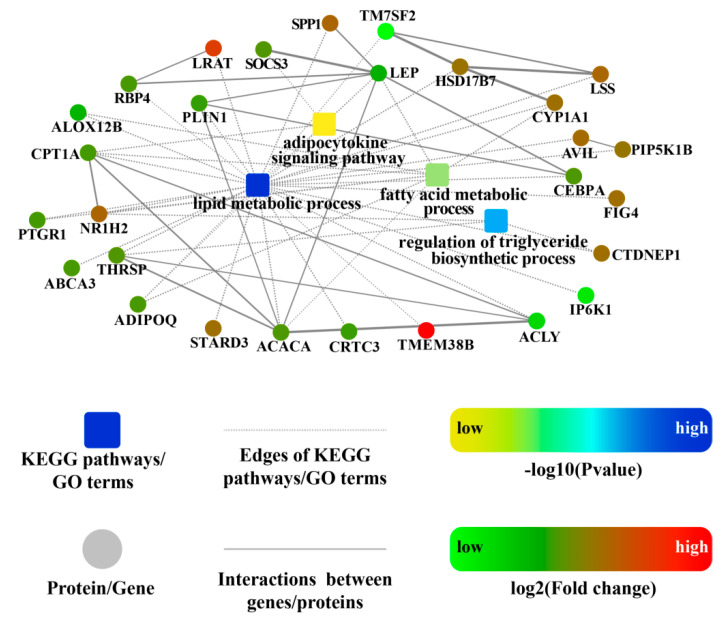
The PPI networks of DEMs in the H vs. L groups. Circle nodes, genes/proteins; rectangle nodes, KEGG pathway or GO terms. Pathways or GO terms are colored with gradient color from yellow to blue, with yellow for smaller *p*-value and blue for bigger *p*-value. According to trend analysis, genes/proteins are colored in red (representing up-regulation) and green (representing down-regulation). Interactions are shown as solid lines between genes/proteins, and edges of KEGG pathways/Go terms are presented as dashed lines.

We then performed a comprehensive bioinformatic analysis of PPI networks among DEMs to further extract relevant information from the identified transcriptome data. As shown in Figure 4, the PPI network of DEMs comprised 32 nodes, 62 edges, and four significantly enriched pathways/terms related to lipid metabolism (the regulation of the triglyceride biosynthetic process, fatty acid metabolic process, lipid metabolic process, and adipocytokine signaling pathway). From this integrated analysis of DEM, GO, and KEGG pathway results, we focused on DEMs that interacted with three or more other genes and were associated with one or more GO/KEGG terms. *LEP*, *ACACA, ACLY*, *PLIN1*, *HSD17B7*, and *CPT1A* were identified as hub genes in the network. Based on these results, we assumed that all of these could be promising candidate genes that affect porcine *longissimus dorsi* fatty acid and lipid metabolism and ultimately the accumulation of IMF.

### 2.3. Expression Patterns of lncRNAs

**Figure 5:**
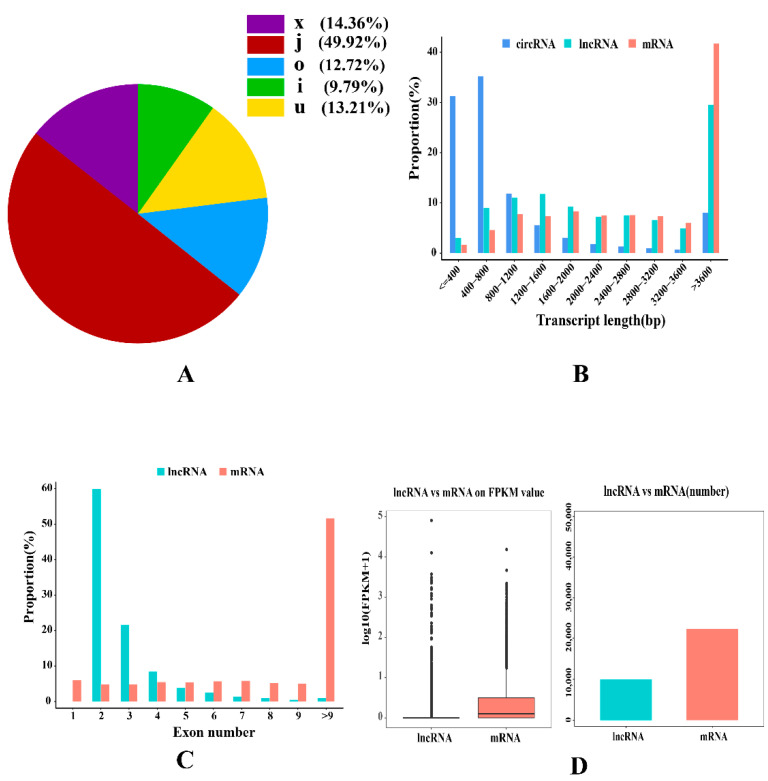
The identification and features of lncRNAs in porcine *longissimus dorsi*. (**A**) Genomic location of lncRNAs. (**B**) Length distribution of lncRNA comparing with circRNAs and mRNA. (**C**) Distribution of exon number for lncRNAs and mRNA. (**D**) Comparison of the expression levels of mRNAs and lncRNAs.

In total, 9980 lncRNAs (including 9367 annotated lncRNAs and 613 novel lncRNAs) were identified on all chromosomes. Five types of lncRNAs were detected, as follows: at least one splice junction shared with a reference transcript (j), a transfrag falling entirely within a reference intron (i), generic exonic overlap with a reference transcript (o), unknown intergenic transcript (u), and exonic overlap with a reference on the opposite strand (x). Most (49.92%) lncRNAs were type j, and the minority (9.79%) were type i (Figure 5A). Most (76.92%) of lncRNAs were more than 1200 bp in length, whereas the length of mRNAs was evenly distributed, ranging from 0 to > 3600 bp (Figure 5B). Compared to protein-coding genes, lncRNAs mostly contained two or three exons (Figure 5C). In addition, the expression levels of lncRNAs were lower than those of protein-coding genes (Figure 5D).

In addition, 91 DE LncRNAs (DELs) were detected in the H vs. L group comparison, with expression levels of 44 lncRNAs upregulated and those of 47 downregulated in the H group (Table 1 and Supplementary File S2). To determine the functions of the identified DELs in IMF deposition, three independent algorithms (antisense, mRNA sequence complementarity; cis, genomic location; and trans, expression correlation) were used to predict the target genes of all DELs. GO and KEGG enrichment analyses were then performed for the target genes. GO analysis revealed that many GO terms related to triglyceride metabolic processes and lipid metabolism were significantly enriched (*p* < 0.05), including the cholesterol metabolic process (GO:0008203), positive regulation of triglyceride metabolic process (GO:0090208), and triglyceride catabolic process (GO:0019433) (Figure 2B, Table 3, and Supplementary File S2). KEGG analysis revealed that these DELs were significantly enriched (*p* < 0.05) in pathways involved in fat and cholesterol metabolism, such as fat digestion and absorption, steroid hormone biosynthesis, sphingolipid metabolism, and cholesterol metabolism (Figure 3B and Supplementary File S3).

### 2.4. Expression Patterns of circRNAs

**Figure 6:**
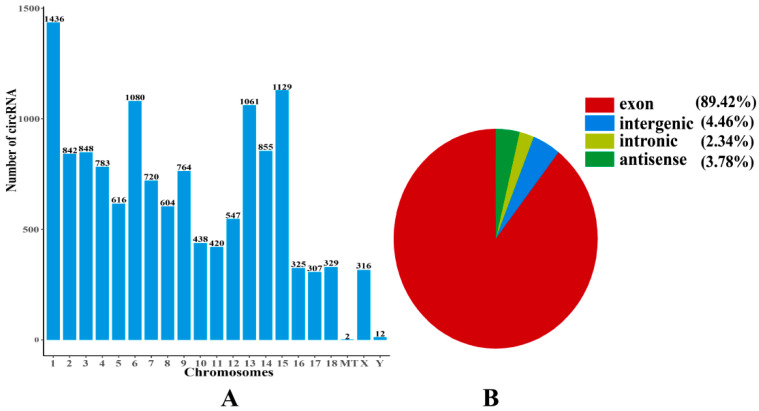
The identification and features of circRNAs in pigs. (**A**) Chromosome distribution of circRNAs. (**B**) Genomic location of circRNAs.

In total, 13,521 circRNAs were identified from all chromosomes. There were 316 and 12 circRNAs detected from the SSC-X and SSC-Y chromosomes, respectively, and two circRNAs were detected from mitochondrial DNA (mtDNA) (Figure 6A). Additionally, four types of circRNAs were identified (Figure 6B) as follows: exon circRNAs (89.42%), intergenic circRNAs (4.46%), antisense circRNAs (3.78%), and intronic circRNAs (2.34%). It has been shown that circRNAs in the *longissimus dorsi* of Yorkshire pigs originate from different genomic regions. Approximately 66.50% of the identified circRNAs were less than 800 bp in length (Figure 5B).

In total, 178 DE CircRNAs (DECs) were identified between the H and L groups, with expression levels of 92 circRNAs upregulated and those of 86 downregulated in the H group (Table 1 and Supplementary File S4). To understand the functional distribution of DECs, functional annotation of DEC parent genes was performed by GO and KEGG pathway analyses. GO analysis revealed that these target genes were significantly enriched (*p* < 0.05) in terms related to lipid metabolism, such as lipid kinase activity (GO:0001727), CoA carboxylase activity (GO:0016421), regulation of lipid kinase activity (GO:0043550), phosphatidylinositol biosynthetic process (GO:0006661), and phosphatidylinositol-translocating ATPase activity (GO:0004012) (Figure 2C, Table 4, and Supplementary File S4). KEGG analysis revealed that these DEC parent genes were significantly enriched (*p* < 0.05) in pathways involved in lipid metabolism, such as purine metabolism and galactose metabolism (Figure 3C and Supplementary File S5).

### 2.5. Detection of miRNA Expression

In total, 36 DE miRNAs (DEMiRs) were defined between the H and L groups, with expression levels of 10 miRNAs downregulated and those of 26 upregulated in the H group (Table 1 and Supplementary File S6). Among them, five DEMiRs have been reported to be related to lipid metabolism, namely ssc-miR-141 (log2FC = 2.908), ssc-miR-192 (log2FC = 2.170), ssc-miR-215 (log2FC = 6.116), ssc-miR-196a (log2FC= −1.295), and ssc-miR-486 (log2FC= −1.007). The function of all DEMiRs target genes was predicted using GO and KEGG pathway analyses. GO analysis revealed that these DEmiRNA-target genes were significantly enriched (*p* < 0.05) in terms related to fat cell differentiation and lipid metabolism, including the fatty acid metabolic process (GO:0006631), fat cell differentiation (GO:0045444), intracellular lipid transport (GO:0032365), regulation of fatty acid oxidation (GO:0046320), and Wnt signaling pathway (GO:0016055) (Figure 2D, Table 5 and Supplementary File S6). KEGG analysis revealed that the target genes were significantly enriched (*p* < 0.05) in pathways that directly regulate adipocyte differentiation, fatty acid biosynthesis, and lipid metabolism, such as the PPAR signaling pathway, MAPK signaling pathway, PI3K-Akt signaling pathway, mTOR signaling pathway, Wnt signaling pathway, biosynthesis of unsaturated fatty acids, fatty acid metabolism, fatty acid elongation, and cholesterol metabolism (Figure 3D and Supplementary File S6).

### 2.6. Construction of the ceRNA Co-Regulatory Network

**Figure 7:**
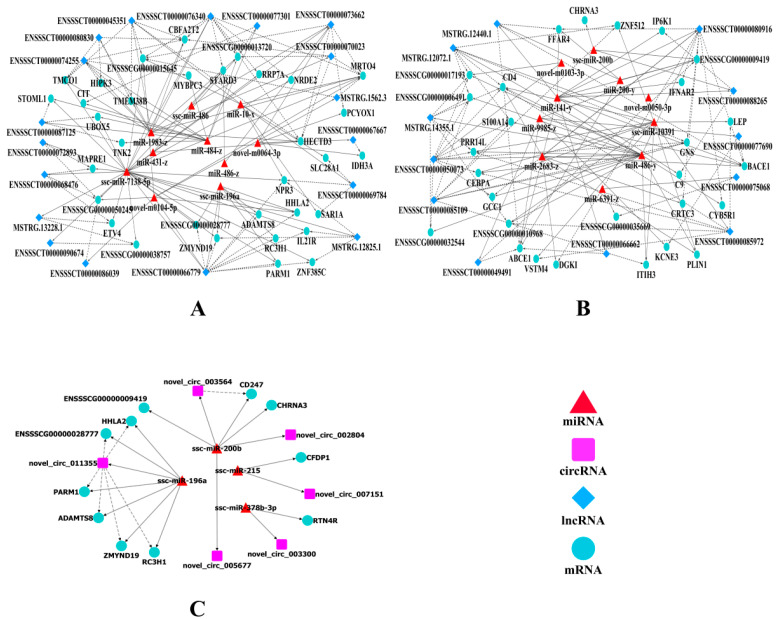
The ceRNA co-regulation networks. Co-regulation networks of lncRNA-miRNA-mRNA (**A**,**B**) and circRNA-miRNA-mRNA (**C**). Triangle points represent miRNAs; Prism points represent lncRNAs; Square points represent circRNAs; Circular point represents mRNAs. The dotted line and the solid line indicate the co-regulation between lncRNAs/circRNAs and mRNAs, and between miRNAs and other transcripts, respectively.

As shown in Figure 7A,B, in the lncRNA–miRNA–mRNA co-regulatory networks, there were 111 nodes and 260 connections among 61 mRNAs, 20 miRNAs, and 30 lncRNAs, and 130 lncRNA–miRNA–mRNA sub network pairs were obtained (Figure 7A,B, Supplementary File S7), such as ENSSSCT00000085972-ssc-miR10391-*LEP*, MSTRG.12825.1-ssc-miR7138-5p-*ZMYND19*, ENSSSCT00000050073-miR486-y-*CEBPA*, ENSSSCT00000066779-ssc-miR-196a-*ADAMTS8*, ENSSSCT00000066779-ssc-miR-7138-5p-*ZMYND19*/*NPR3*, ENSSSCT00000066779-ssc-miR-196a-*ZMYND19*/*PARM1*, ENSSSCT00000067667-novel-m0064-3p-*IDH3*,ENSSSCT00000080916-miR-486-y-*CYB5R1*, ENSSSCT00000080916-miR-2683-z-*DGKI*, ENSSSCT00000080916-miR-2683-z-*CRTC3*, ENSSSCT00000088265-miR-141-y/ssc-miR-200b-*IP6K1*, ENSSSCT00000076340-ssc-miR7138-5p-*STARD3*, and MSTRG.12440.1-miR-141-y-*FFAR4*. The circRNA–miRNA–mRNA co-regulatory network (Figure 7C) included 21 nodes and 23 connections among 11 mRNAs, four miRNAs, and five circRNAs. Seven circRNA–miRNA–mRNA sub-network pairs were also obtained (Supplementary File S7), including novel_circ_011355-ssc-miR-196a-*ADAMTS8* and novel_circ_011355-ssc-miR-196a-*ZMYND19*/*PARM1*. These results indicate that lncRNAs and circRNAs can regulate gene expression by acting as miRNA sponges, suggesting that *LEP*, *ZMYND19*, *CEBPA*, *ADAMTS8*, *ZMYND19*, *IDH3*, *CYB5R1*, *DGKI*, *CRTC3*, *IP6K1*, *FFAR4*, and *STARD3*, as well as other genes related to fatty acid and lipid metabolism, might be crucial genes modulated by non-coding RNAs that regulate IMF deposition.

### 2.7. qRT-PCR Validation

**Figure 8:**
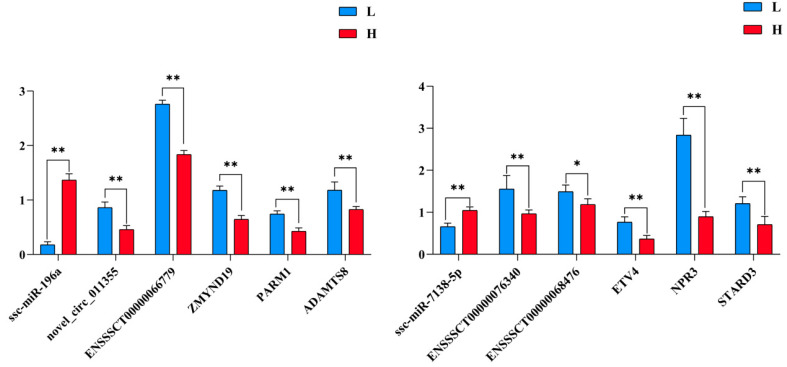
The expression patterns of ssc-miR-196a, ssc-miR-7138-5p, related target genes were verified by qRT-PCR. The data represented the Mean ± SD from 5 biological replicates, and each measurement was repeated 3 times. ** indicates *p* < 0.01, * indicates *p* < 0.05.

To validate potential interactions in the networks, the expression levels of ssc-miR-196a and ssc-miR7138-5p, related ceRNAs, were measured by qRT-PCR. The expression of ssc-miR-196a was upregulated in the H group. In contrast, that of its target genes, including novel_circ_011355, ENSSSCT00000066779, *ZMYND19*, *PARM1*, and *ADAMTS8* was downregulated (Figure 8). The expression level of ssc-miR7138-5p was downregulated in the H group. In contrast, that of its target genes, including ENSSSCT00000066779, ENSSSCT00000076340, ENSSSCT00000068476, *ETV4*, *NPR3*, and *STARD3*, was upregulated (Figure 8). In addition, validation of the RNA-seq results was carried out using qRT-PCR for another randomly selected 15 genes, including two DEMs (*CHRNA3* and *TMEM38B*), five DECs (novel_circ_002804, novel_circ_008940, novel_circ_001557, novel_circ_011588, novel_circ_003997), four DEMiRs (ssc-miR-200b, miR-1983-z, miR-486-y, miR-10-x), and four DELs (ENSSSCT00000080712, ENSSSCT00000090965, MSTRG.12825.1, ENSSSCT00000070023). The expression patterns of these transcripts were highly consistent with those obtained by RNA-seq (Table S4), indicating the high reproducibility and reliability of the gene expression profiles obtained in this study.

## 3. Discussion

During the last decade, meat producers have started to focus more on pork quality. The IMF content, which represents the amount of fat, including phospholipids, triglycerides, and cholesterol within muscles, is an important factor that is positively associated with overall meat quality [20,21]. Pork with a higher IMF tends to be of better quality, resulting in higher overall acceptability [22]. Given the importance of IMF in the economics of pig meat production, clarifying the molecular mechanisms underlying IMF deposition in pigs is of great significance. In the present study, longissimus dorsi whole-transcriptomes of two groups of Yorkshire purebred finishing castrated boars divergent in IMF content were comprehensively analyzed to compare differences in the expression profiles of mRNAs and non-coding RNAs. This was performed to identify candidate genes and pathways related to the divergent IMF deposition and to interpret the complex molecular cascade events related to the variability observed in this trait. In addition, potential non-coding RNA–miRNA–mRNA co-expression ceRNA networks were constructed and preliminarily validated, providing new insights into the molecular mechanisms of porcine IMF deposition.

Comparing gene expression between individuals with divergent traits in the same population can reduce noise owing to different genetic backgrounds [12]. In the present study, all pigs were selected based on the same breed, sex, and weight to allow candidate genes to be more dependable, thereby excluding unreliable factors identified owing to different breeds or different feed conditions. Yorkshire pigs are a typical lean-type western breed that has been intensively selected over the past few decades to increase lean meat production and reduce fat deposition; however, they still exhibit considerable phenotypic variation in fatty traits in the population [16]. Too much intrapopulation variation is a disadvantage for commercial production [4]. Moreover, Yorkshire breeds often serve as the first dam line in crossbred (Duroc × (Landrace × Yorkshire)) pig production [23], the first sire line in crossbred (Duroc × (Yorkshire × Landrace)) pig production [24], and the sire line in the binary hybridization production of Chinese local pigs [25], which means that they contribute approximately one-third or one-half of the genetic material passed on to the offspring. Therefore, it will be beneficial and efficient to genetically improve the IMF of Yorkshire pigs. Because of the particular characteristics of the Yorkshire pig, it is particularly interesting to analyze changes in the transcriptome and regulatory networks of finishing Yorkshire pigs with divergent IMF content.

In total, 341 DEMs were identified from the sequencing data, of which the expression levels of 207 DEMs were upregulated in the H group, including a series of lipogenic genes, such as *ACACA*, *ACLY*, *PTGR1*, *DGAT2*, and *CYB5R1*. These differentially expressed genes are involved in various aspects of de novo fatty acid synthesis, including direct and indirect regulation, and play catalytic roles in fatty acid biosynthesis [18,26,27,28]. Moreover, the expression differences in *ACACA*, *ACLY*, *PTGR1*, *DGAT2*, and *CYB5R1* between the H and L groups were consistent with the increasing IMF deposition trend (Supplementary File S1), indicating that these genes might act as positive regulators of the IMF deposition process. Furthermore, many DEMs (*LEP*, P*IP5K1B*, *SREBF2*, *CPT1A*, *ALOX12B*, *IP6K1*, *NR1H2*, *ACBD7*, *DGKI*, *SPP1*, *CYP1A1*, *LPIN1*, *HSD17B7*, *FFAR4*, *ADIPOQ*, *ADAMTS8*, and *HSD11B1*) were significantly enriched in fatty acid biosynthesis and lipid metabolism-related GO terms and KEGG pathways. Among these genes, *LEP* encodes a protein, leptin, primarily secreted by white adipocytes but also expressed in other tissues including skeletal muscle, was also reported to be associated with porcine *longissimus dorsi* IMF content [29]. Individuals with a high body fat composition have higher levels of leptin [30]. In our trial, *LEP* expression tended to be upregulated in the H group, which agrees with the typical fat composition of this group when compared to that of the L group. The *ADIPOQ* gene encodes a protein hormone, adiponectin, which is secreted by adipocytes and is involved in the regulation and inhibition of lipogenesis and the stimulation of fatty acid oxidation [31]. Consistent with our results, the expression of *ADIPOQ* was higher in Lantang, a high-IMF pig breed, than in Landrace, a low-IMF pig breed [32]. Meanwhile, *HSD17B7* encodes an enzyme involved in cholesterol biosynthesis [33], whereas *PLIN1* belongs to the periplasmin family of proteins and is associated with the formation of intracellular lipid droplets [34]. Further, *CPT1A* encodes carnitine palmitoyltransferase-1, an enzyme responsible for transporting long-chain fatty acids for Î²-oxidation. In our trial, *CPT1A* expression was upregulated in pigs with a high IMF content, which is consistent with a previous report showing that its expression level is positively correlated with IMF content [35]. Interestingly, in our PPI network results, we identified six hub genes, including *LEP*, *ACACA*, *ACLY*, *PLIN1*, *HSD17B7*, and *CPT1A*, most of which are involved in fatty acid and lipid metabolism. These results suggest that genes responsible for fatty acid and lipid metabolism in the *longissimus dorsi* tissue significantly differ between high- and low-IMF Yorkshire pigs.

In this study, 91 DELs were identified from sequencing data, including 60 known lncRNAs and 31 novel lncRNAs. To further investigate the difference in IMF deposition between H and L groups, we predicted the target genes of these DELs (Supplementary File S8). Some of these differentially expressed lncRNA-target genes have been reported to be important for IMF deposition. *LPL* (lipoprotein lipase), the target gene of ENSSSCT00000050073, is involved in fatty acid catabolism, and its expression level is positively associated with swine IMF content [36]. The *PPARG* gene for MSTRG.14355.1, encoding the core regulator of the PPAR signaling pathway, regulates lipid metabolism and glucose homeostasis, promotes adipocyte differentiation and fat deposition, and harbors polymorphisms that could significantly affect IMF deposition in pigs [37]. *HMGCR*, the target gene of ENSSSCT00000072909, and MSTRG.4737.2, a cholesterol-synthesis-limiting enzyme, harbor one polymorphism that shows a positive relationship with IMF in pigs [38]. Moreover, the target genes of other DELs (Supplementary File S8), including ENSSSCT00000066793, ENSSSCT00000069743, ENSSSCT00000077515, MSTRG.14478.1, ENSSSCT00000080216, MSTRG.1567.1, MSTRG.4080.9 (target gene: *ACACA*), ENSSSCT00000074683, ENSSSCT00000083654, ENSSSCT00000084825, ENSSSCT00000085428, ENSSSCT00000085972, ENSSSCT00000089741, ENSSSCT00000089741, MSTRG.14434.8, MSTRG.14355.1 (target gene: *LEP*), ENSSSCT00000066499, MSTRG.10478.1, MSTRG.232.1 (target gene: *ACLY*), ENSSSCT00000086039 (target gene: *DGAT2*), ENSSSCT00000050073, ENSSSCT00000088265, MSTRG.12072.1, MSTRG.14478.1 (target gene: *PTGR1*), ENSSSCT00000073662, ENSSSCT00000075506, ENSSSCT00000087125, and MSTRG.5410.1 (target gene: *SREBF1*), are also closely related to fat metabolism [29,39]. KEGG and GO analyses revealed that many fat and cholesterol metabolism-related pathways and terms were significantly enriched, and some genes related to lipid metabolism were enriched multiple times, including *APOA1*, *APOA4*, *APOB*, *MTTP*, *ABCG5*, and *ABCG8* [40,41,42,43,44]. Furthermore, via trans prediction, we found that ENSSSCT00000062623, ENSSSCT00000069577, ENSSSCT00000069784, ENSSSCT00000075507, ENSSSCT00000077869, MSTRG.129.2, and MSTRG.5755.1 all formed targeting relationships with *APOA1*, *APOA4*, *APOB*, *MTTP*, *ABCG5*, and *ABCG8* (Supplementary File S8), and the expression levels of these seven DELs were all higher in the high IMF group, suggesting that these DELs might facilitate lipid transport and metabolism. Based on these results, we suspected that one of the main roles of these lncRNAs is to regulate IMF deposition in pigs, and further highlighting their detailed mechanisms in this process would provide a strong basis for future investigations.

CircRNAs have been found to play important roles in adipogenesis and lipid metabolism [45,46]. In this study, we identified 178 DECs. The functions of circRNAs are assumed to be related to those of their host genes. Some of these DEC host genes have been reported to be important for fat deposition (Supplementary File S4). *LRP6*, the host gene of novel_circ_000878, is a well-established factor that governs lipid generation and secretion, which regulates body fat and glucose homeostasis by modulating nutrient-sensing pathways and mitochondrial energy expenditure [47]. *PLIN1*, the host gene of novel_circ_011588, plays an important role in regulating lipolysis and lipid storage in adipocytes and is reported to be a candidate gene affecting porcine IMF content [48]. Furthermore, KEGG and GO analyses revealed that some lipid metabolic process-related pathways and terms were significantly enriched, and some host genes were enriched multiple times, including *ALOX15* (novel_circ_006541), *PDGFA* (novel_circ_005676), *DAB2IP* (novel_circ_008787), and *PCYT1A* (novel_ circ_009957). *ALOX15*, a lipoxygenase isoform, is involved in the metabolism of linoleic and arachidonic acids [49]. *DAB2IP* (novel_circ_008787), a Ras GAP, regulates lipid droplet homeostasis by acting as a GAP for *RAB40C* [50]. Further, *PCYT1A* (novel_ circ_009957) is the major isoform of the key enzyme CTP (choline phosphate cytidylyltransferase), which is essential for phosphatidylcholine synthesis during lipid metabolism [51]. *PDGFA* (novel_circ_005676) plays a vital role in the proliferation and maintenance of adipocyte progenitors in dermal adipose tissue through the PI3K–Akt pathway [52] and was found to be a potential candidate marker that regulates pig growth and fat deposition [53]. Meanwhile, in the GO analyses, glycerolipid biosynthetic process (GO:0045017) and arachidonate 12-lipoxygenase activity (GO:0004052) were significantly enriched for both DEMs and DECs; further, some GO terms were also enriched for both DEMs and DECs but only significantly enriched for one of them, such as the lipid metabolic process (GO:0006629) and regulation of lipid localization (GO:1905952) (Supplementary File S4). These results indicate that the differences in lipid metabolism and fat deposition between the high- and low-IMF pigs are regulated not only by mRNAs but also by circRNAs.

miRNAs have been reported to play important roles in the regulation of preadipocyte differentiation and fat deposition [16,54,55]. In this study, 36 DE miRNAs were defined between the H and L groups. Among them, ssc-miR-141, ssc-miR-215, ssc-miR-196a, and ssc-miR-486 have been reported to be related to preadipocyte differentiation and adipogenesis in pigs [56,57,58,59]. Novel DEMiR-target genes have also been reported to be important for lipid metabolism and fat deposition (Supplementary File S9), including novel-m0048-3p (*LEP*, *LEPR*, *LPL*, *CYB5R1*, *VLDLR*, *CPT1A*, *PPARD*), novel-m0050-3p (*ACACA*, *PLIN1*, *THRSP*), and novel-m0104-5p (*FFAR4*, *PPARA*, *APOE*) [60,61,62]. Furthermore, KEGG and GO analyses revealed that the target genes of 36 DEMiRs were significantly enriched for terms and pathways related to fat cell differentiation, fatty acid biosynthesis, and lipid metabolism, such as the PPAR signaling pathway [63], PI3K–Akt signaling pathway [64], mTOR signaling pathway [65], and Wnt signaling pathway [66].

Studies have shown that mRNAs, lncRNAs, and circRNAs regulate the expression of each other (when sharing the same miRNA-binding sites) by functioning as competing endogenous RNAs (miRNA sponges) during adipocyte differentiation. For example, the lncRNA ADNCR inhibits adipocyte differentiation by functioning as a ceRNA for miR-204, thereby augmenting expression of the miR-204-target gene *SIRT1* [67]. Further, circFLT1 and lncCCPG1 sponge miR-93 to regulate the proliferation and differentiation of adipocytes by promoting lncSLC30A9 expression [68]. In this study, combined with the co-differentially expressed DEMs, DELs, DECs, and DEMiRs, we constructed ceRNA regulatory networks, which showed that 30 lncRNAs and 61 mRNAs exhibited crosstalk with each other through 20 miRNAs, and that five circRNAs and 11 mRNAs showed crosstalk through four miRNAs. Consistent with previous studies [19,69], this also indicates that IMF deposition in pigs results from a balance in gene expression. Furthermore, from the ceRNA networks, we observed a series of ceRNA subnetworks that might play key roles in the regulation of IMF deposition. For example, MSTRG.12440.1 and its target *FFAR4* exhibited crosstalk through miR-141-y and miR-200-y response elements, ENSSSCT00000085972 and its target *LEP* showed crosstalk through miR-10391, ENSSSCT00000050073 and its target CEBPA exhibited crosstalk through miR486-y, and novel_circ_011355 and its targets *PARM1*, *ZMYND19,* and *ADAMTS8* showed crosstalk through miR-196a. *FFAR4*, *LEP*, *PARM1*, *CEBPA*, *ZMYND19*, and *ADAMTS8* have been reported to be involved in fat metabolism [70,71,72]. In addition, several nodes were found to be shared by both lncRNA–miRNA–mRNA co-regulatory networks and circRNA–miRNA–mRNA co-regulatory networks, namely ssc-miR-196a, *ZMYND19*, *PARM1*, and *ATAMTS8*. From these data, it could be inferred that the identified non-coding RNAs participate in the intramuscular adipogenesis process by acting as ceRNAs and that the genes involved in ceRNA regulatory networks might play an important role in IMF deposition through molecular synergism and the upregulation of important pathways, which should be studied in the future.

## 4. Materials and Methods

### 4.1. The Experimental Animals and Sample Collection

A Yorkshire finishing pig resource population was housed at Anhui Lvjian Breeding Pig Co., Ltd. (Quanjiao, China) under consistent and standard environmental conditions. In total, 75 healthy castrated males of similar ages and weights (approximately 170-days-old and 125 kg, respectively) were selected. The live backfat thickness was measured 5 cm from the left dorsal midline between the last third and fourth ribs using real-time B-mode ultrasonography. All 75 live backfat thickness phenotypic values were fitted normal distribution. The two tails of the distribution, including 5 samples for each, were established by two investigation groups. According to these values, five pigs with extremely high (16.66 ± 0.78 mm) and five pigs with extremely low (9.14 ± 0.74 mm) live backfat thickness were slaughtered in the same batch. After slaughter, the average three-point backfat thicknesses of carcasses at the shoulder end of the dorsal midline, the thoracolumbar junction, and the lumbar spine junction were measured using vernier calipers as the carcass backfat thickness. Samples of *longissimus dorsi* muscle at the 3rd/4th last rib were collected [73], with a portion of the samples stored at −20 °C for IMF determination and the rest frozen in liquid nitrogen until RNA isolation. The IMF was measured as a percentage using the Soxhlet extraction method [74]. The average IMF values were 7.18% (SD = ±0.013) and 1.64% (SD = ±0.006) in the high and low live backfat thickness groups, respectively. The carcass backfat thickness values were 38.20 mm (SD = ±1.29) and 18.06 mm (SD = ±1.42) for these groups, respectively. The significance of the differences in these three fatness traits between the two groups was assessed using a *t*-test in SPSS 20.0, with all *p* values < 0.001. Subsequently, three individuals were randomly selected from the extremely high and low live backfat thickness, IMF, and carcass backfat thickness groups to comprise the extremely high IMF (H) and low IMF (L) groups for RNA-seq analysis.

### 4.2. mRNA/lncRNA/circRNA Sequencing and Data Analysis

Total RNA was isolated from each longissimus dorsi sample (six individuals) using the TRIzol reagent kit (Invitrogen, Carlsbad, CA, USA). After the integrity, purity, and quality of the isolated RNA were tested, samples with an RNA integrity number greater than seven were used for further analysis. Ribosomal RNA (rRNA) was removed from the DNA-free RNA using the Ribo-ZeroTM kit (Epicenter, Madison, WI, USA). DNA and rRNA-free RNA were used to create a library using the NEBNext UltraTM RNA Library Prep Kit for Illumina (NEB, E7530L). The library was sequenced using an Illumina Novaseq6000 instrument (Gene Denovo Biotechnology Co., Guangzhou, China). Raw data from the six pig transcriptomes were uploaded to the NCBI SRA under the accession number PRJNA821451.

Clean data were obtained by filtering the adapters, unknown bases, and low-quality reads. HISAT2 (2.10) [75] was used to map the clean reads to the Sus scrofa 11.1 reference genome (http://ftp.ensembl.org/pub/release-103/fasta/sus_scrofa/ (accessed on 2 May 2021)). Gene abundance was quantified with RSEM [76]. The fragments per kilobase of transcript per million (FPKM) value was then used to represent the expression levels of the genes.

### 4.3. Identification of lncRNAs

Novel transcripts were reconstructed using StringTie software (v1.3.4) [77,78] with default parameters using the mapped clean reads, and GffCompare was used to screen known mRNAs and other non-coding RNAs (rRNA, tRNA, snoRNA, snRNA, known lncRNA, etc.). Furthermore, known lncRNAs were identified through comparative analysis. Subsequently, novel potential lncRNA transcripts were identified according to the transcript length (>200 bp) and exon number (≥2). Then, CNCI (v2.0) [79] (score < 0) and CPC (v0.9-r2) [80] (score < 0) were used to predict the protein-coding potential of the transcripts. Finally, the intersection of both non-protein-coding potential results were chosen as novel lncRNAs.

### 4.4. Identification of circRNAs

After the clean reads were aligned to the porcine reference genome, junctions of the unmapped reads were identified using a back-splice algorithm. Findcirc software (v1.0) [81] was used to predict circRNAs. The expression levels of circRNAs were reflected by the number of mapped back-splicing junction reads per million mapped reads.

### 4.5. Small RNA Library Construction and Sequencing

Total RNA from each longissimus dorsi sample (six individuals) was isolated. RNA quality and quantity were determined using a Bioanalyzer 2100 (Agilent Technologies, Palo Alto, CA, USA). The adapters were then added, and the 36–48 nt RNAs were enriched by polyacrylamide gel electrophoresis. The final products were amplified using reverse transcription (RT)-PCR to construct a cDNA library. They were then sequenced using an Illumina Novaseq6000 by Gene Denovo Biotechnology Co. (Guangzhou, China). Raw miRNA sequencing data were uploaded to the NCBI Biotechnology Information database (PRJNA824228).

### 4.6. Alignment and Identification of Small RNA

To obtain clean tags, raw reads were further filtered, as with conventional processing [82]. All clean tags were aligned with small RNAs in the GenBank database (Release 209.0) and the Rfam database (Release 11.0) to identify and remove rRNA, scRNA, snoRNA, snRNA, and tRNA. All clean tags were also aligned to the reference genome to remove tags mapped to exons, introns, or repeat sequences. Next, the filtered tags were searched against the miRBase database (Release 22) to identify the known porcine miRNAs. The unannotated tags were predicted to be and identified as novel miRNAs using mirdeep2 software, according to the tag positions in the genome and their hairpin structures.

### 4.7. Prediction of Target Genes of miRNAs

Miranda (v3.3a) and TargetScan (v7.0) were used to predict miRNA targets. The intersection of the results was selected as the predicted miRNA-target genes.

### 4.8. Differentially Expressed RNA Identification and Pathway Analysis

DESeq2 software [83] was used to detect differentially expressed mRNAs (DEMs), circRNAs (DECs), miRNAs (DEMiRs), and lncRNAs (DELs), with values of *p* < 0.05 and |log2fold-change (FC)| ≥ 1. Gene Ontology (GO) term and the Kyoto Encyclopedia of Genes and Genomes (KEGG) analysis were performed for DELs (using the DEL target genes, which were predicted in a cis, trans, and antisense manner), DEMiRs (using the DEMiR target genes), DECs (using the DEC parent genes), and DEMs using the DAVID tool, and *p* < 0.05 was considered significant.

### 4.9. Protein–Protein Interaction (PPI) Analysis of Differentially Expressed mRNAs

PPI analysis of DEMs was performed using the STRING database (http://string-db.org/cgi/input.pl (accessed on 30 July 2022)), which is a well-known tool used to predict PPIs. The following network model was generated based on information gained from up to four levels of functional analysis: fold-change of genes or proteins, PPIs, KEGG pathway enrichment, and GO enrichment. DEMs were mapped to STRING with a confidence score > 0.4, which is a reasonable score used to limit the number of interactions to those with higher confidence that are much more likely to be true positives. PPI networks were generated using Cytoscape software (v3.3.0) [84].

### 4.10. Construction of the lncRNA/circRNA–miRNA–mRNA Network

To reveal the functions of and interactions among ncRNAs and mRNAs, we constructed an ncRNA–mRNA regulatory network. The ceRNA network was constructed based on ceRNA theory. The prediction of target genes for differentially expressed miRNAs was the first step. The mRNA–miRNA, lncRNA–miRNA, or circRNA–miRNA expression correlations were evaluated using the Spearman rank correlation coefficient (SCC). Pairs with an SCC value < −0.7 were selected as negatively co-expressed lncRNA–miRNA, mRNA–miRNA, or circRNA–miRNA pairs. mRNA, lncRNA, and circRNA were miRNA-target genes, and all RNAs were differentially expressed. The lncRNA–mRNA and circRNA–mRNA expression correlations were evaluated using the Pearson correlation coefficient (PCC). Pairs with a PCC value > 0.9 were selected as co-expressed lncRNA–mRNA pairs or circRNA–mRNA pairs. Both the mRNA and lncRNA in such pairs were targeted and negatively co-expressed with a common miRNA or both mRNA and circRNA in such pairs were targeted and negatively co-expressed with a common miRNA. The ceRNA regulatory network was constructed by assembling all co-expressed competing triplets, which were identified previously herein and visualized using Cytoscape software (v3.3.0) [84].

### 4.11. qRT-PCR

First, based on the lncRNA/circRNA–miRNA–mRNA network, 10 nodes around ssc-miR-196a and ssc-miR-7138-5p, including three lncRNAs (ENSSSCT00000066779, ENSSSCT00000076340, and ENSSSCT00000068476), one circRNA (novel_circ_011355), and six mRNAs (*ZMYND19*, *PARM1*, *ADAMTS8*, *ETV4*, *NPR3*, and *STARD3*) were selected. Two other DEMs, five DECs, four DEMiRs, and four DELs were also randomly selected. The expression trends in the H and L groups were validated using qRT-PCR. The RNA used in the validation experiment was that of pigs with extremely high (n = 5) and low (n = 5) live backfat thickness, IMF, and carcass backfat thickness.

The primers used are listed in Supplemental Table S1. Glyceraldehyde-3-phosphate dehydrogenase (*GAPDH*; for mRNA, lncRNA, and circRNA) and U6 (for miRNA) were used as endogenous controls. The synthesized cDNA was used as a template for RT-qPCR using a CFX96 TouchTM Real-Time PCR Detection System (Bio-Rad, Hercules, CA, USA). All reactions were performed in triplicate for each sample. The 2^−∆∆CT^ method was used to quantify changes in relative gene expression. Significant differences were analyzed by a t-test using SPSS 22.0 with the following criteria: *p* < 0.05 (*), *p* < 0.01 (**).

## 5. Conclusions

The present study provides a comprehensive understanding of the differences in the whole-transcriptome profiles of longissimus dorsi tissues between high- and low-IMF Yorkshire finishing pigs. In summary, 341 DEMs, 91 DELs, 178 DECs, and 36 DEMiRs were characterized and found to be widely involved in terms and pathways related to fatty acid metabolic processes, lipid metabolism, and fat cell differentiation, indicating that non-coding RNAs are abundant in the longissimus dorsi muscle and are involved in porcine adipocyte adipogenesis and lipid metabolism. Furthermore, we constructed ceRNA regulatory networks and found a series of ceRNA subnetworks that might play a key role in the regulation of IMF deposition. Specifically, the results showed that functional lncRNAs and circRNAs (such as MSTRG.12440.1, ENSSSCT00000066779, ENSSSCT00000076340, ENSSSCT00000050073, MSTRG.12825.1, ENSSSCT00000085972, ENSSSCT00000080916, and novel_circ_011355, novel_circ_011355) act as ceRNAs of important fat deposition-related mRNAs (*LEP*, *ZMYND19*, *CEBPA*, *ADAMTS8*, *ZMYND19*, *IDH3*, *CYB5R1*, *DGKI*, *CRTC3*, *IP6K1*, *FFAR4*, and *STARD3*, etc.) by sponging functional miRNAs (such as ssc-miR-196a, ssc-miR-200b, ssc-miR10391, miR486-y, ssc-miR7138-5p, and novel-m0064-3p, miR-2683-z). These results provide a strong foundation for future investigations, as well as potential regulators and molecular regulatory networks for in vitro and in vivo experiments to study the detailed mechanisms underlying IMF deposition in pigs. This work also has an important significance for the pig industry, especially for breeding pigs based on the IMF trait.

## Figures and Tables

**Table 1:** The number of DE mRNAs, lncRNAs, and circRNAs in the H vs. L groups.

Terms	UR mRNAs	DR mRNAs	UR lncRNAs	DR lncRNAs	UR circRNAs	DR circRNAs	UR miRNAs	DR miRNAs
H vs. L	207	134	44	47	92	86	26	10

Note: UR, Up-regulated; DR, Down-regulated.

**Table 2:** A statistical gene ontology (GO) analysis of DEMs between the H and L groups (only a part of terms related to lipid metabolism are listed).

GO ID	Description	*p* Value	Genes
0048871	multicellular organismal homeostasis	0.0001	*COL11A2*; *NR1H2*; *TRIM32*; *ABCA3*; *FFAR4*; *RBP4*; *IP6K1*; *ACACA*; *ALOX12B*; *COL2A1*; *PBLD*; *DLL1*; *CRTC3*; *NPR3*; *ADIPOQ*; *LEP*; *DIO2*
0032368	regulation of lipid transport	0.0001	*FURIN*; *NR1H2*; *ABCA3*; *LRAT*; *SPP1*; *SREBF2*; *ADIPOQ*; *LEP*
0006629	lipid metabolic process	0.0002	*PLIN1*; *CYP1A1*; *CEBPA*; *NR1H2*; *FIG4*; *HSD17B7*; *ABCA3*; *LRAT*; *SPP1*; *RBP4*; *IP6K1*; *CPT1A*; *TM7SF2*; *ACLY*; *STARD3*; *ACACA*; *CTDNEP1*; *ALOX12B*; *PTGR1*; *LSS*; *PIP5K1B*; *AVIL*; *THRSP*; *CRTC3*; *ADIPOQ*; *TMEM38B*; *LEP*
0050873	brown fat cell differentiation	0.0004	*CEBPA*; *FFAR4*; *ADIPOQ*; *LEP*; *DIO2*
1905952	regulation of lipid localization	0.0004	*FURIN*; *NR1H2*; *ABCA3*; *LRAT*; *SPP1*; *SREBF2*; *ADIPOQ*; *LEP*
0042445	hormone metabolic process	0.0004	*FURIN*; *CYP1A1*; *HSD17B7*; *LRAT*; *SPP1*; *RBP4*; *STARD3*; *LEP*; *DIO2*
0032371	regulation of sterol transport	0.0005	*FURIN*; *NR1H2*; *SREBF2*; *ADIPOQ*; *LEP*
0032374	regulation of cholesterol transport	0.0005	*FURIN*; *NR1H2*; *SREBF2*; *ADIPOQ*; *LEP*
0034754	cellular hormone metabolic process	0.0006	*CYP1A1*; *HSD17B7*; *LRAT*; *SPP1*; *RBP4*; *STARD3*
0008610	lipid biosynthetic process	0.0009	*NR1H2*; *HSD17B7*; *ABCA3*; *TM7SF2*; *ACLY*; *STARD3*; *ACACA*; *CTDNEP1*; *ALOX12B*; *LSS*; *PIP5K1B*; *AVIL*; *THRSP*; *TMEM38B*; *LEP*
0010866	regulation of triglyceride biosynthetic process	0.0012	*NR1H2*; *CTDNEP1*; *THRSP*
0046460	neutral lipid biosynthetic process	0.0012	*NR1H2*; *CTDNEP1*; *AVIL*; *THRSP*
0046463	acylglycerol biosynthetic process	0.0012	*NR1H2*; *CTDNEP1*; *AVIL*; *THRSP*
0106106	cold-induced thermogenesis	0.0013	*NR1H2*; *FFAR4*; *IP6K1*; *NPR3*; *ADIPOQ*; *LEP*; *DIO2*
0120161	regulation of cold-induced thermogenesis	0.0013	*NR1H2*; *FFAR4*; *IP6K1*; *NPR3*; *ADIPOQ*; *LEP*; *DIO2*

**Table 3:** A statistical gene ontology (GO) analysis of DELs between the H and L groups (only a part of the terms related to lipid metabolism are listed).

GO ID	Description	*p* Value	Genes
0044241	lipid digestion	0.0006	*ABCG8*; *ENSSSCG00000010432*; *NPC1L1*; *APOA1*; *LIMA1*; *ENSSSCG00000033190*; *ABCG5*; *LEP*; *APOA4*
0098856	intestinal lipid absorption	0.0019	*ABCG8*; *NPC1L1*; *APOA1*; *LIMA1*; *ABCG5*; *FABP2*; *LEP*; *APOA4*
0042953	lipoprotein transport	0.0137	*APOBEC1*; *APOB*; *MTTP*; *PPARG*; *UNC119*
0034370	triglyceride-rich lipoprotein particle remodeling	0.0213	*NR1H4*; *APOA1*; *LPL*; *APOA4*
0055088	lipid homeostasis	0.0240	*NR1H4*; *MYLIP*; *NR1H2*; *ANGPTL3*; *LIPG*; *LIPC*; *HNF4A*; *ABCA3*; *ABCG8*; *APOB*; *MTTP*; *LAMTOR1*; *ITGB6*; *ABCB11*; *MED13*; *ACACA*; *NUS1*; *SOAT1*; *SREBF2*; *APOA1*; *RTN4*; *LIMA1*; *ABCG5*; *LPL*; *APOA4*
0019433	triglyceride catabolic process	0.0261	APOB; PNLIPRP2; PNPLA3; AADAC; LPL; APOA4
0008203	cholesterol metabolic process	0.0308	*ANGPTL3*; *LIPC*; *GBA2*; *HSD17B7*; *APOB*; *NSDHL*; *TM7SF2*; *HMGCR*; *CFTR*; *NPC1L1*; *LSS*; *SOAT1*; *CLN8*; *ULT2B1*; *CYP11A1*; *SCAP*; *CEL*; *APOA1*; *SREBF1*; *PIP4P1*; *LEP*; *APOA4*
0030301	cholesterol transport	0.0353	*FURIN*; *NR1H2*; *LIPG*; *LIPC*; *RELCH*; *ABCG8*; *APOB*; *PIP4K2A*; *LAMTOR1*; *CFTR*; *NPC1L1*; *NUS1*; *SOAT1*; *STX12*; *SREBF2*; *APOA1*; *STARD4*; *LIMA1*; *ABCG5*; *LEP*; *APOA4*
0006084	acetyl-CoA metabolic process	0.0366	*ACSS1*; *PDK1*; *ACLY*; *ACACA*; *PIPOX*; *ACOT12*; *HMGCS2*
0090208	positive regulation of triglyceride metabolic process	0.0399	*NR1H2*; *MFSD2A*; *CTDNEP1*; *AADAC*; *SREBF1*; *APOA4*
0016406	carnitine O-acyltransferase activity	0.0304	*CRAT*; *CPT1A*; *CROT*
0106018	phosphatidylinositol-3,5-bisphosphate phosphatase activity	0.0304	*MTM1*; *MTMR2*; *MTMR6*
0005319	lipid transporter activity	0.0370	*SLC5A8*; *STRA6*; *MFSD2A*; *ENSSSCG00000005307*; *ABCA3*; *ABCG8*; *APOB*; *MTTP*; *PITPNB*; *SLC51A*; *ABCB11*; *ANO9*; *APOA1*; *STARD4*; *ABCC11*; *ABCG5*; *GLTP*; *APOA4*
0034040	lipid-transporting ATPase activity	0.0433	*ABCB11*; *ABCC11*
0042627	chylomicron	0.0298	*APOB*; *APOA4*; *APOH*

**Table 4:** A statistical gene ontology (GO) analysis of DECs between the H and L groups (Only a part of terms related to lipid metabolism are listed).

GO ID	Description	*p* Value	Genes
1903726	negative regulation of phospholipid metabolic process	0.0037	*DAB2IP*; *PDGFA*
0006644	phospholipid metabolic process	0.0039	*DAB2IP*; *PDGFA*; *PCYT1A*; *AMBRA1*; *PIK3C2B*; *ALOX15*; *SMG1*
0046474	glycerophospholipid biosynthetic process	0.0043	*PDGFA*; *PCYT1A*; *PIK3C2B*; *ALOX15*
1903725	regulation of phospholipid metabolic process	0.0107	*DAB2IP*; *PDGFA*; *AMBRA1*
0036314	response to sterol	0.0108	*LRP6*; *RORA*
0008654	phospholipid biosynthetic process	0.0108	*PDGFA*; *PCYT1A*; *PIK3C2B*; *ALOX15*
0045017	glycerolipid biosynthetic process	0.0112	*PDGFA*; *PCYT1A*; *PIK3C2B*; *ALOX15*
0006650	glycerophospholipid metabolic process	0.0139	*PDGFA*; *PCYT1A*; *PIK3C2B*; *ALOX15*; *SMG1*
0046488	phosphatidylinositol metabolic process	0.0267	*PDGFA*; *PIK3C2B*; *SMG1*
0043553	negative regulation of phosphatidylinositol 3-kinase activity	0.0278	*DAB2IP*
2001300	lipoxin metabolic process	0.0278	*ALOX15*
0006661	phosphatidylinositol biosynthetic process	0.0445	*PDGFA*; *PIK3C2B*
0010511	regulation of phosphatidylinositol biosynthetic process	0.0459	*PDGFA*
0019321	pentose metabolic process	0.0459	*FGGY*
0019372	lipoxygenase pathway	0.0459	*ALOX15*

**Table 5:** A statistical gene ontology (GO) analysis of DEMiRs between the H and L groups (Only a part of terms related to lipid metabolism are listed).

GO ID	Description	*p* Value	Gene Number
0006631	fatty acid metabolic process	0.0026	139
0045444	fat cell differentiation	0.0040	126
0046486	glycerolipid metabolic process	0.0070	161
0050994	regulation of lipid catabolic process	0.0091	27
0032365	intracellular lipid transport	0.0136	15
0046890	regulation of lipid biosynthetic process	0.0171	79
0046320	regulation of fatty acid oxidation	0.0192	14
0016055	Wnt signaling pathway	0.0229	199
0030497	fatty acid elongation	0.0259	9
0050873	brown fat cell differentiation	0.0269	29
0006633	fatty acid biosynthetic process	0.0288	53
0019395	fatty acid oxidation	0.0298	45
1905952	regulation of lipid localization	0.0364	74
0034440	lipid oxidation	0.0401	46
0006635	fatty acid beta-oxidation	0.0476	32

## Data Availability

Raw data from the six pig transcriptomes were uploaded to the NCBI SRA (https://submit.ncbi.nlm.nih.gov/) under the accession number PRJNA821451. Raw miRNA sequencing data were uploaded to the NCBI (https://submit.ncbi.nlm.nih.gov/) Biotechnology Information database (PRJNA824228).

## Supplementary Materials

The following supporting information can be downloaded at: https://www.mdpi.com/article/10.3390/ijms232012596/s1.

## Abbreviations

CircRNA: Circular RNA; LncRNA: Long non-coding RNA; MiRNA: MicroRNA; mRNA: Messenger RNA; FPKM: Fragments per kilobase of transcript per million reads mapped; CNCI: Coding-non-coding index; CPC: Coding potential calculator; GO: Gene ontology; KEGG: Kyoto Encyclopedia of Genes and Genomes; RNA-Seq: RNA sequencing; rRNA: Ribosomal RNA; SnoRNA: Small nucleolar RNA; SnRNA: Small nuclear RNA; qRT-PCR: Quantitative real-time RT-PCR; DE: differentially expressed; DEMs: differentially expressed mRNAs; DECs: differentially expressed circRNAs; DELs: differentially expressed lncRNAs; DEMiRs: differentially expressed miRNAs; ceRNA: competing endogenous RNA; ncRNA: Non-coding RNAs; IMF: Intramuscular fat; H: high; L: low; PPI: Protein–protein Interaction; SCC: Spearman rank correlation coefficient; PCC: Pearson correlation coefficient.
