# Supplementary material for: Identification of Candidate Genes and Regulatory Competitive Endogenous RNA (ceRNA) Networks Underlying Intramuscular Fat Content in Yorkshire Pigs with Extreme Fat Deposition Phenotypes
Source: Int J Mol Sci. 2022 Oct 20;23(20):12596. doi: 10.3390/ijms232012596 (PMC9603960; doi:10.3390/ijms232012596)
Supplement: Supplementary file 1 [file ijms-23-12596-s001.zip › TableS1 The primers used for qRT-PCR.pdf]

Table S1 Primer sequences for qRT-PCR

Type	Gene name	Primer sequences(5'-3')
circRNA	novel_circ_001557	F: ACTGTGTCCTTTATACCCTTCCTG
		R: TGTTCTTCCCCTATAGTCTTCTTAAAG
	novel_circ_002804	F: GGTGAAAGACAACCCAAAGCC
		R: GTTCAGCCAAGCATCCTTCTG
	novel_circ_011588	F: CTCGTACCCTAATTCCGACAGAG
		R: GGATTCAGCCCCTTGTAGAGC
	novel_circ_008940	F: CCTCGTAAACAAGCCACCCAG
		R: CCCATTCGCCATCTACAGGAC
	novel_circ_003997	F: CAGAAACAGAGGAGTTGGAGTTGC
		R: TTTTTGTCGAGATGAGGAACTGG
	novel_circ_011355	F: TGTTCCTGCTTGGCTAAATTTCTC
		R: ACGGAGTTAACGGAAGGTAGTGAG
lncRNA	MSTRG.12825.1	F: CAGAGTTCCCAATGCCAAATG
		R: CATCTTGAGTTTCAGTGCTGTGC
	ENSSSCT00000076340	F: CCTTACGTTTTCTCAGGTCAGTCC
		R: CATGGTTGCCATGCATCTCC
	ENSSSCT00000070023	F: AGTTAAAATCCCTCTCGTCCTGAC
		R: TGTTTGCATCAAGACCGCTTC
	ENSSSCT00000090965	F: GTACAGAGATGTGATGCTGGAGAAC
		R: AGAAAACCGATCACACTCTAGGC
	ENSSSCT00000066779	F: GACAAGGTGTGCTGTGGAATCTC
		R: GGTCAGGGTGTGAATGTCAGATG
	ENSSSCT00000080712	F: AACACTGAATCCTGAAGCCTATTTG
		R: ACCTTCGAGAGGTACAGAGTTTCAG
	ENSSSCT00000068476	F: TCTTCCTAAACAGATACTTACCTCCC
		R: CCTGTTCTGTATGACCCAGTGAG
mRNA	ZMYND19	F:GAAGAGGAGGAGACGTCCTG
		R: GCCCACAGATGTTGAACTCC
	PARM1	F: TGTCAGTCCTGGCAAACTCT
		R: TGTGACGATGAAGAGAGCGA
	ADAMTS8	F: TCCTGGAGTTACCAGCAAGG
		R: ACTGCTCTTTGGAAGCTCCT
	CHRNA3	F: TTCAACCGTACCGAGTCCAA
		R: AGCATGGCGTCAACAGATTC
	ETV4	F: GCCCTCTTCTCTCTGGCTTT
		R: CAAAGGGACTGTGTCCTCCT
	STARD3	F: TCGACCTGCTCTTCATCTCC
		R: AGGCCAGGACAAAGATGTCA
	NPR3	F: TTTACAGCGACGACAAGCTG
		R: CCAAGCCTTTGGTCTCATCG
	TMEM38B	F: CTGCCAAGGTAACCCTGTTG
		R: AAACAGCATCCGACTCAACG

control	GAPDH	F: ATCACTGCCACCCAGAAGACTG<br>R: GCCAGTGAGCTTCCCGTTG
miRNA	ssc-miR-196a	StemLoopPrimer:GTCGTATCCAGTGCAGGGTCCGAGGTATTCGCACTGGATACGACCCCAAC<br>F: CGCGCGTAGGTAGTTTCATGTT<br>R: AGTGCAGGGTCCGAGGTATT
	ssc-miR-7138-5p	StemLoopPrimer:GTCGTATCCAGTGCAGGGTCCGAGGTATTCGCACTGGATACGACAGATGG<br>F: CGTCCCAGCAAGTGTCCAT<br>R: AGTGCAGGGTCCGAGGTATT
	ssc-miR-200b	StemLoopPrimer:GTCGTATCCAGTGCAGGGTCCGAGGTATTCGCACTGGATACGACGTCATC<br>F: GCGCGTAATACTGCCTGGTAAT<br>R: AGTGCAGGGTCCGAGGTATT
	miR-10-x	StemLoopPrimer:GTCGTATCCAGTGCAGGGTCCGAGGTATTCGCACTGGATACGACTCACAA<br>F: GCGACCCTGTAGAACCGAAT<br>R: AGTGCAGGGTCCGAGGTATT
	miR-1983-z	StemLoopPrimer:GTCGTATCCAGTGCAGGGTCCGAGGTATTCGCACTGGATACGACAGAAAA<br>F: GCGCTCACCTGGAGCATG<br>R: AGTGCAGGGTCCGAGGTATT
	miR-486-y	StemLoopPrimer:<br>GTCGTATCCAGTGCAGGGTCCGAGGTATTCGCACTGGATACGACGGTCCT<br>F: CGCGGGGCAGCTCAGTAC<br>R: AGTGCAGGGTCCGAGGTATT
control	U6	StemLoopPrimer:<br>GTCGTATCCAGTGCAGGGTCCGAGGTATTCGCACTGGATACGACAAAAATATGG<br>F: GCTCGCTTCGGCAGCACATATAC<br>R: AGTGCAGGGTCCGAGGTATT
