# Supplementary material for: Identification of Candidate Genes and Regulatory Competitive Endogenous RNA (ceRNA) Networks Underlying Intramuscular Fat Content in Yorkshire Pigs with Extreme Fat Deposition Phenotypes
Source: Int J Mol Sci. 2022 Oct 20;23(20):12596. doi: 10.3390/ijms232012596 (PMC9603960; doi:10.3390/ijms232012596)
Supplement: Supplementary file 1 [file ijms-23-12596-s001.zip › TableS2 Overview of the data for RNA-seq.pdf]

Table S2 Overview of the data for RNA sequencing

Sample	H1	H2	H3	L1	L2	L3
Total reads	82,783,190	74,674,250	125,164,208	90,922,804	128,176,402	138,483,666
Clean reads	82,402,852	74,367,466	124,581,402	90,505,406	12,780,608	137,892,896
Celan ratio, %	99.54	99.59	99.53	99.54	99.71	99.57
Mapped reads	77,955,285	71,263,260	118,996,799	86,574,028	119,848,395	131,868,944
Mpped ratio, %	94.94	95.94	95.69	95.86	93.86	95.76
Unique mapped reads	70,411,832	63,809,261	108,236,953	79,049,259	111,896,495	120,832,800
Unique ratio, %	85.76	85.90	87.04	87.53	87.63	87.74
GC content, %	55.91	56.05	55.48	55.54	53.68	55.64
Q30, %	93.05	93.76	93.70	93.71	94.11	93.43
