# Supplementary material for: Identification of Candidate Genes and Regulatory Competitive Endogenous RNA (ceRNA) Networks Underlying Intramuscular Fat Content in Yorkshire Pigs with Extreme Fat Deposition Phenotypes
Source: Int J Mol Sci. 2022 Oct 20;23(20):12596. doi: 10.3390/ijms232012596 (PMC9603960; doi:10.3390/ijms232012596)
Supplement: Supplementary file 1 [file ijms-23-12596-s001.zip › TableS3 Overview of the data for small RNA-seq.pdf]

Table S3 Overview of the data for small RNA sequencing

Sample	H1	H2	H3	L1	L2	L3
Total reads	13,048,848	9,990,814	9,959,318	9,295,100	11,516,326	13,349,696
Clean reads	12,734,725	9,747,637	9,685,832	9077,898	11,189,782	13,004,202
Celan ration, %	97.59	97.57	97.25	97.66	97.16	97.41
known_mirna reads	798,070	641,092	688,117	738,085	605,135	800,110
novel_mirna reads	8,785	7,116	8,052	6,648	7,042	10,156
exist_ reads	9,471,102	7,085,021	7,028,516	6,286,823	8,488,455	9,535,926
known_mirna number	472	487	499	479	511	481
novel_mirna number	157	146	153	150	140	158
exist_mirna number	330	324	328	322	332	325
