# Supplementary material for: Identification of Candidate Genes and Regulatory Competitive Endogenous RNA (ceRNA) Networks Underlying Intramuscular Fat Content in Yorkshire Pigs with Extreme Fat Deposition Phenotypes
Source: Int J Mol Sci. 2022 Oct 20;23(20):12596. doi: 10.3390/ijms232012596 (PMC9603960; doi:10.3390/ijms232012596)
Supplement: Supplementary file 1 [file ijms-23-12596-s001.zip › TableS4 The qRT-PCR Results.pdf]

Table S4 qRT-PCR Results

Type	Gene name	RNA-seq result H-vs-L Log2（Fold Change）	qRT-PCR result H-vs-L Log2（Fold Change）	P value	Confirmed result of sequence
CeRNA	ssc-miR-196a	-1.3	-2.85	<0.0001	Yes
	novel_circ_011355	1.96	0.91	<0.0001	Yes
	ZMYND19	-3.81	-0.59	<0.0001	Yes
	PARM1	1.94	0.87	<0.0001	Yes
	ADAMTS8	7.54	0.8	<0.0001	Yes
	**ENSSSCT00000066779**	2.92	0.51	0.0012	Yes
	ssc-miR-7138-5p	-0.94	-0.66	<0.0001	Yes
	ENSSSCT00000076340	-1.94	-0.69	0.0042	Yes
	ENSSSCT00000068476	-6.32	-0.34	0.011	Yes
	ETV4	1.61	1.07	0.0004	Yes
	NPR3	3.15	1.49	<0.0001	Yes
	STARD3	2.16	0.77	0.0021	Yes
CircRNA	novel_circ_002804	-1.92	-0.39	<0.0001	Yes
	novel_circ_008940	-16.36	-1.62	0.0005	Yes
	novel_circ_001557	-16.55	-0.62	<0.0001	Yes
	novel_circ_011588	16.51	1.18	0.0005	Yes
	novel_circ_003997	16.55	1.4	<0.0001	Yes
LncRNA	ENSSSCT00000080712	6.06	0.81	<0.0001	Yes
	ENSSSCT00000090965	2.49	0.49	<0.0001	Yes
	MSTRG.12825.1	-1.13	-0.67	0.002	Yes
	ENSSSCT00000070023	-1.47	-1.6	<0.0001	Yes
miRNA	ssc-miR-200b	2.69	1.14	<0.0001	Yes
	miR-1983-z	-1.13	-2.8	<0.0001	Yes
	miR-486-y	1.66	1.21	0.0021	Yes
	miR-10-x	0.04	-1.49	0.0009	Yes
mRNA	CHRNA3	-4.64	-1.02	0.001	Yes
	TMEM38B	11.36	0.75	0.0034	Yes
